# How does a video feedback intervention work for people with dementia and distress behaviour living in a nursing home, for whom and under which circumstances? A realist interview study

**DOI:** 10.1016/j.ijnsa.2026.100523

**Published:** 2026-03-16

**Authors:** Stephanie Timmermans, Annette Plouvier, Anne Kolmans, Mandy Wijnen, Anke Persoon, Annemiek Bielderman, Cécile Boot, Debby Gerritsen

**Affiliations:** a Research Institute for Medical Innovation, Radboud university medical center, Postbus 9101, 6500 HB Nijmegen, the Netherlands; b Department of Primary and Community care, Radboud university medical center, p/a Radboudumc, afd. ELG-149, Postbus 9101, 6500 HB Nijmegen, the Netherlands; c University Knowledge Network for Older adult care Nijmegen (UKON), Radboud university medical center, p/a Radboudumc, afd. ELG-149, Postbus 9101, 6500 HB Nijmegen, the Netherlands; d Radboudumc Alzheimer Center, Radboud university medical center, p/a Radboudumc, afd. 925, Postbus 9101, 6500 HB Nijmegen, the Netherlands; e Amsterdam UMC, VU University, Department of Public and Occupational Health, Postbus 7057, 1007MB Amsterdam, the Netherlands; f Radboud University, Behavioural Science Institute, Postbus 9104, 6500 HE Nijmegen, the Netherlands

**Keywords:** Distress behaviour, Dementia, Nursing homes, Realist evaluation, Video feedback intervention

## Abstract

**Background:**

Distress behaviour of people living with dementia is associated with negative effects for the person concerned, their family, and care staff. Video feedback interventions seem promising to use in nursing homes in cases of distress behaviour. In the Netherlands, a video feedback intervention “VIO” (Video Interventie Ouderenzorg) – in which the care team reflectively watches video footage, is asked stimulating questions by the VIO counsellor, and practises with assignments – can be used. This study aims to explain how VIO works, for whom, and under which circumstances.

**Methods:**

A purposive sample of eight VIO counsellors and three care team members was interviewed in this realist interview study conducted in the Netherlands in 2022. The data was analysed by building Context-Mechanism-Outcome configurations.

**Results:**

Participants reported that VIO can improve verbal and nonverbal communication with the person living with dementia and can work because team members become aware of their contribution to the interaction with that person and change their behaviour accordingly. According to the participants the impact of VIO can be determined by different factors, such as safety and trust within the care team, having a team member with an exemplary role, trust between the VIO counsellor and the care team, as well as by organisational conditions, such as the lack of staff, high sick leave, and managerial support. The role of family was rarely mentioned.

**Conclusions:**

The design of VIO aligns with the way in which the care team learns. The intended outcomes of improved verbal and nonverbal communication and a decrease in distress behaviour are more likely to be achieved when team’s and organisational conditions, and the VIO counsellor’s approach are satisfactory. Future research should focus on testing and refining the discovered Context-Mechanism-Outcome configurations in practice. For daily practice, it is recommended to explore how to overcome incorrect expectations, and how to increase the role of family.

**What is already known**

• Distress behaviour is reported to have negative effects for those involved.

• Video feedback can improve communication, and thus influence distress behaviour.

**What this paper adds**

• VIO can work because care staff become aware of their contribution.

• Team, organisational and counsellor’s factors are experienced to have an influence.

• The current role of family in VIO when applied in nursing homes seems limited.

Alt-text: Unlabelled box dummy alt text

## 1 Introduction

Globally, the number of people affected by dementia is growing (G. B. D. Dementia Forecasting Collaborators, 2022; Guerchet et al., 2020). As dementia progresses, distress behaviour tends to become more prevalent (Helvik et al., 2018; Ostaszkiewicz et al., 2015). Distress behaviour such as agitation, apathy and aggression is associated with numerous negative effects. For the person living with dementia, distress behaviour can contribute to reduced quality of life (Klapwijk et al., 2018). Moreover, the distress experienced by family members as a result of this behaviour is a frequent reason for admission to a nursing home (Afram et al., 2014). Furthermore, distress resulting from this behaviour can also be experienced by nursing home care staff (Hazelhof et al., 2016; Ostaszkiewicz et al., 2015).

It has been recognised that the behaviour of care staff influences the behaviour of people living with dementia (Haunch et al., 2022; Helgesen et al., 2010; Veldwijk-Rouwenhorst et al., 2022). Moreover, it is known that interactions can become more difficult when behaviour of people living with dementia is misunderstood (Cameron et al., 2020; Volicer and Hurley, 2003). Adequate skills of nurses, such as empathy, compassion, and communication skills are therefore crucial (Scott et al., 2011; Veldwijk-Rouwenhorst et al., 2022). Likewise, the results of Piirainen et al. (2021) suggest therapeutic use of self and social competence to be among the most important competencies in caring for people living with dementia. Video feedback interventions seem promising to support the adequate interpretation of the behaviour of the person with dementia and to attune to it. Video feedback intervention is an approach to learning in which interactions are recorded and watched back later. It provides caregivers with the opportunity to re-evaluate their own interpretation of the behaviour of the person living with dementia, and closely watch how an interaction between the person living with dementia and themselves or a peer progresses (Hansebo and Kihlgren, 2001; O'Rourke et al., 2020).

Although interventions differ in their design, the few studies focusing on video feedback in long-term dementia care show promising results (O'Rourke et al., 2020). For example, a quasi-experimental study into video feedback in Norwegian dementia care units found that the intervention group, consisting of dyads of a nurse and a person living with dementia, scored higher on supporting communicative elements than the control group (Alnes et al., 2011). This suggests video feedback interventions have the potential to improve communication between caregivers and the person with dementia. Furthermore, in a Norwegian action research study in a dementia care unit, video feedback counselling shifted the focus from generalised skills to contextual and relational care, allowing the care team to learn together (Lykkeslet et al., 2016). This is supported by a Canadian pilot study in a nursing home, the results of which suggest that video feedback intervention promotes person-centred communication (O'Rourke et al., 2022). Nurses interviewed about their experiences with video feedback intervention reported acquiring knowledge both about the person living with dementia and about themselves, and awareness of how their own actions could influence interactions with the person living with dementia (Einang Alnes et al., 2011).

The results of these studies suggest that video feedback interventions have positive effects. To our knowledge, however, much is still unknown about how, for whom and under which circumstances video feedback interventions for people living with dementia and distress behaviour work in nursing homes. Our aim is to contribute to this knowledge hiatus by investigating how, for whom and under which circumstances a Dutch video feedback intervention called “VIO” works.

## 2 Methods

### 2.1 Design

A realist evaluation was conducted, using realist interviews. Realist evaluation is based in a realist philosophy of science called ‘scientific realism’ (Pawson, 2006) and therefore belongs to neither the positivist nor constructivist paradigm. Realism’s focus is to explain how and why complex programs work, for whom, under which circumstances (Jagosh, 2019; Pawson, 2013), whilst acknowledging that these interventions are implemented in complex reality, which is not always fully observable. Moreover, realism views knowledge generated through evaluations as an approximation of this “real world” (Manzano, 2016). A study will never result in an “end-solution”, but rather in refined theory about how and why an intervention works. Retroduction is the required analytical strategy, and realism has its own terminology and quality standards (Wong et al., 2017), focusing on explanatory adequacy. The five main terms will be explained in the box below (Jagosh, 2019; Jagosh et al., 2015; Manzano, 2022; van der Weide et al., 2025; van Hees et al., 2022).

Box 1 Definition of realist terms.

*Context:* Elements in the intervention that impact the outcomes by enabling or disabling a specific mechanism to “fire”.

*Mechanism:* Resources the programme offers and the (often internal/implicit) responses of people to those resources.

*Outcome:* (un)Intended consequences following from context-mechanism interactions.

*Context-Mechanism-Outcome configuration:* Heuristic used in realist evaluation to focus on deep generative causation by linking context, mechanism, and outcome.

*Retroduction:* Reasoning to develop or refine theory by moving backwards from outcomes to explanations of what may have led to the outcomes.

Alt-text: Unlabelled box dummy alt text

### 2.2 Intervention VIO

**Fig. 1:**
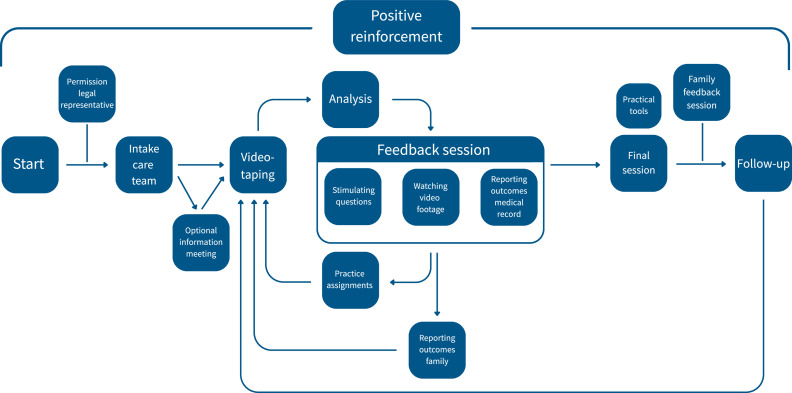
A chronological depiction of VIO.

VIO is an existing intervention, that is part of care as usual. The aim of VIO in nursing homes is to improve interactions and attunement between the person living with dementia and the care team, as well as care team’s competencies to respond to distress behaviour. These are stepping stones for improving the care relationship between the person living with dementia and the care team. The underlying idea is that the competencies of the care team members can grow by learning together and from each other, and by gaining awareness of their contribution to the interaction. A decrease of the distress behaviour of the person living with dementia in frequency and severity can be an additional effect, but this is not an explicit aim of VIO. A chronological depiction of VIO can be found in Fig. 1.

There is a strict protocol on how VIO is performed. VIO starts with asking permission of the legal representative of the person with dementia to use VIO, followed by an intake in which the VIO counsellor discusses the care situation and the expectations and objectives of VIO with 2–3 members of the care team. Afterwards, a moment of interaction between a team member and the person with dementia is videotaped and first analysed by the VIO counsellor. If the VIO counsellor notices distress in the person with dementia during videotaping, recording will stop. In a subsequent feedback session with the entire care team, specific snippets of this videotaping - selected by the counsellor based on the counsellor’s analysis - are discussed, focusing on understanding the person living with dementia. Each feedback session results in practice assignments made by the counsellor together with the care team and every step is reported in the patient portfolio and to family. This process of videorecording, analysis, feedback session, and practice assignment is repeated 3–5 times, and a trajectory lasts several weeks. VIO concludes with a final multidisciplinary feedback session in which all recordings are discussed once more and practical tools are presented, that can be incorporated in the care plan. After that session has taken place, the family is invited to watch all recordings of the trajectory in one single session with the counsellor. As a follow-up, the VIO counsellor contacts the care team 6 months after VIO is finished. If the change brought about by VIO is not long lasting, the VIO counsellor can schedule an extra feedback session. Throughout this entire process the VIO counsellor ensures a focus on positive reinforcement.

### 2.3 Setting and participants

This study was conducted between May and June 2022 in the Netherlands. As realist evaluation uses the criteria of relevance and rigour to control for quality, rather than saturation, no number of interviews was determined a priori (Manzano, 2016). Instead we focused on selecting interviewees who could help us theorise how, for whom and under which circumstances VIO works (relevance), and on collecting the data in a credible, trustworthy, and plausible way (rigour) (Hunter et al., 2022; van der Weide et al., 2023). Taking into account the goal of this study, it was important to start theorising with those who are most knowledgeable and experienced with the intervention: VIO counsellors (Manzano, 2016). Although VIO counsellors were the main focus of this study, we deemed it necessary to include the care staff’s perspective as well. In line with realist pragmatism (Manzano, 2016), we decided to recruit care staff members with ample experience with VIO.

Purposive sampling was used as the sampling technique for VIO counsellors (Patton, 2014; Thijssen et al., 2023). Their recruitment took place through the VIO expertise centre located in the south of the Netherlands and through the Centre for Consultation and Expertise, which employs VIO counsellors all through the Netherlands. VIO counsellors were approached by members of the project team, who shared information on the study, and asked if the VIO counsellors were willing to participate in the study. If the potential interviewees answered affirmative, their contact information would be shared with the researcher, who would send an information letter on the study. After the interviewees had the chance to ask questions, an appointment was set for the interview. Informed consent was obtained and a demographic questionnaire was sent to the interviewees, in order for the interviewer to prepare the interview.

Care staff were selected by the VIO counsellors of the VIO expertise centre who had already taken part in the study through a snowball sampling approach. The VIO counsellors shared information on the study and asked care staff members if they were willing to participate. Otherwise, the informed consent procedure was identical to that of the VIO counsellors.

### 2.4 Data collection

The data were collected through face-to-face or online realist interviews (ST). The interviews were semi-structured and revolved around a topic guide with open-ended questions based on grey and scientific literature of the intervention (Gerritsen, Koopmans, et al., 2019), realist principles (Westhorp and Manzano, 2017), and discussion and refinement within the project team. The topic guide was the same for all interviewees and started with five questions on what possible outcomes VIO has, such as ‘Can you tell me what outcomes result from working with VIO for the person with dementia and distress behaviour?’. This was followed by five questions on which mechanisms could cause and explain these outcomes, such as ‘How do you think VIO brings about these outcomes?’. The last part of the topic guide considered how different contexts could influence the working mechanisms, and thereby the outcomes of VIO, with four questions such as ‘To what degree does the success of VIO depend on the organisation that VIO is employed within?’. This order was chosen because we expected it to benefit the retroductive thinking in the interviewee (Meyer and Lunnay, 2013; Mukumbang et al., 2018).

The interviews lasted approximately 60 min each. All interviews were audio recorded with the permission of the interviewees, pseudonymised, and transcribed verbatim.

### 2.5 Data analysis

The data was analysed by using the heuristic of the realist evaluation framework, namely Context-Mechanism-Outcome configuration. Two researchers (ST, AK) read through each transcript three times to immerse themselves in the data, whereafter the most relevant passages were exported into a report (rigour and relevance). As a next step in the analysis process, Context-Mechanism-Outcome were configured (ST, AK) and aggregated at the mechanism level (ST, AK, AP) based on the reports (Jackson and Kolla, 2012). This was an iterative process in which information from additional reports was compared against already created Context-Mechanism-Outcome configurations. This either led to Context-Mechanism-Outcome refinement or a new Context-Mechanism-Outcome configuration (explanatory adequacy). After that, the entire project team (ST, AP, AK, MW, AP, AB, CB, DG) was involved in linking the aggregated Context-Mechanism-Outcome configurations, grouping them into topics, based on mechanism, and visualising these into a model of the process of VIO through multiple rounds of iteration. As a final step, this model and the included Context-Mechanism-Outcome configurations were checked for accuracy with two VIO experts.

### 2.6 Ethical considerations

This study was performed in agreement with the Declaration of Helsinki. Through a preliminary assessment by the Medical Ethics Review Committee (CMO Arnhem-Nijmegen region) in December 2021, it was determined that the study was not subject to the Medical Research Involving Human Subjects Act (decision number 2021–13,410). All interviewees gave written informed consent and their privacy rights have been observed.

## 3 Results

Interviews were held with eight VIO counsellors and three care team members. Nine of the interviewees were female. The mean age was 53.8 (*SD* = 8.6, range: 38–66). The working experience in dementia care averaged 14.1 years (*SD* = 6.4, range: 5–25), the experience of the VIO counsellors with VIO averaged 10.4 years (*SD =* 6.5, range: 2–17). The occupations of the care team members were nurse or certified nurse assistant. The interviewees were equally divided between the expertise centre (six) and the national consultation centre (five). The last time the interviewees were involved in a VIO trajectory differed and ranged from ‘at the moment’ to 10 months ago.

Seven topics concerning how, for whom, and under which circumstances VIO works were identified. Pertaining the care team, these were learning styles and motivation; safety and trust within the team; team members with an exemplary role; and expectations of VIO. The other three topics concerned the VIO counsellor’s personal approach, organisational conditions and the role of the family of the person living with dementia. Below, we will elaborate on each topic, illustrated by several quotes and at least one Context-Mechanism-Outcome configuration.

**Fig. 2:**
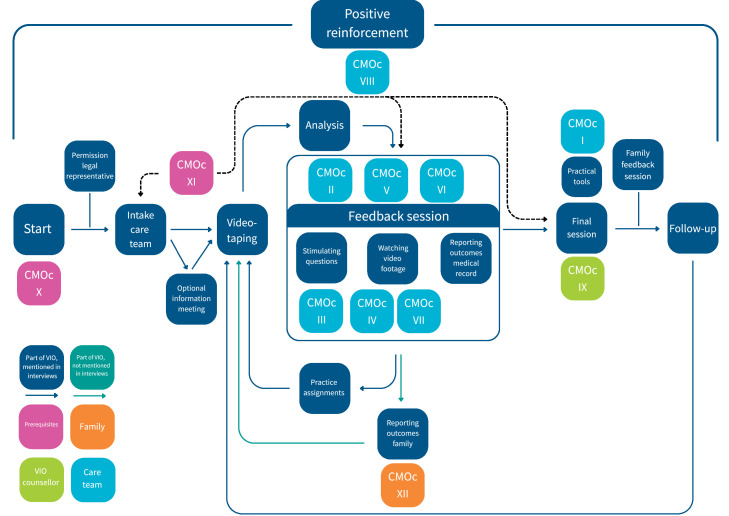
A visualisation of the Context-Mechanism-Outcome configurations within VIO.

Fig. 2 visualises the Context-Mechanism-Outcome configurations in the model of VIO. It gives an overview of the role the different topics play at the different stages of VIO, resulting in different outcomes.

### 3.1 Care team

Four topics pertaining to the care team were identified, which will be discussed below in the following order: Learning styles and motivation, Safety and trust within the team, Team members with an exemplary role and Expectations of VIO.

#### 3.1.1 Learning styles and motivation

Interviewees described several aspects pertaining to learning and motivation. First, they felt that the practical nature of VIO was effective in changing the care team’s approach of the person with dementia, because it is very much attuned to the learning style of the care team, much more than written advice.

*“Elderly care employees often learn from doing, you should not give them lots of written information. […] If you visualise (the information), make it practical, it’s just more effectively retained.”* (Interviewee 5, VIO counsellor)

Another interviewee described how lessons learned during VIO could reach further than just that specific situation or person living with dementia that was subject of the video footage.

*“It really brings out your strengths and you can utilise those deliberately at other times or with other residents.”* (Interviewee 11, certified nurse assistant)

The practical tools, in which lessons learned during the intervention were incorporated, were integrated in the care plan to further facilitate retention.

*“That’s the most important, learning to see what happens, acting accordingly and using the practical tools you get offered to deal with it.”* (Interviewee 2, nurse)

Context-Mechanism-Outcome configuration I

In line with the manner in which care employees learn (context), practical tools resulting from VIO, help team members in recognising and predicting similar situations in the future (mechanism). This improves verbal and nonverbal communication between team members and the person living with dementia (outcome), saves time (outcome), and improves job satisfaction of the team members (outcome). Similarly, tools resulting from VIO help team members in applying the lessons in other situations (mechanism), which improves verbal and nonverbal communication with other people living with dementia as well (outcome).

A team member illustrated how understanding the person with dementia led to more attuned care and a change in appreciation.

*“You just start seeing a resident differently. Someone that seems impossible to work with in the beginning, and through VIO and getting practical tools you think ‘what a fantastic person she actually is […] It became very clear that she needs clarity. For example, don’t say ‘we will go for a walk in a moment’ but ‘we will go for a walk at two o’clock’.”* (Interviewee 2, nurse)

Context-Mechanism-Outcome configuration II

In line with the manner in which care employees learn (context), the focus of the feedback sessions on understanding the person living with dementia, makes team members realise who the person with dementia is (mechanism). This leads to improved verbal and nonverbal communication between team members and the person living with dementia (outcome), decreased distress behaviour and crisis situations (outcome), an improved relationship between team members and person living with dementia (outcome), improved quality of care (outcome), and improved quality of life of the person with dementia (outcome).

Several VIO counsellors additionally elaborated on the essential role of asking stimulating questions during the feedback session.

*“I can tell them (the care team) what I want them to focus on, but that doesn’t work very well. They have to discover themselves ‘Hey, I do this’ or ‘We see this happening’ and then you ask ‘why is that important? What effect do you see in the resident?’ They have to feel responsible and experience ownership of what is being put forward, that works best.”* (Interviewee 4, VIO counsellor)

Context-Mechanism-Outcome configuration III

The stimulating questions asked by the VIO counsellor during the feedback sessions evoke feelings of responsibility in team members (mechanism), especially if the team members are already aware of their own role in the interactions (context). This leads to team members expressing their desire to change the current situation (outcome), improved verbal and nonverbal communication between team members and the person with dementia (outcome), and a feeling of increased control over the situation by team members (outcome).

Finally, reflective watching was mentioned by both VIO counsellors and team members as an explanation of why VIO works.

*“If you are delivering care, you perceive the situation very differently compared to when you are looking at an on-screen recording. Using that, you see a shift in attribution. People (at first) place it (the cause of the difficult interaction) outside themselves. Slowly, they also start to look inwards, ‘What is my contribution? What can I change?’”* (Interviewee 7, VIO counsellor)

Context-Mechanism-Outcome configuration IV

In line with the manner in which care employees learn (context), looking at the video footage during feedback sessions, guides the team members to look at interactions reflectively (mechanism). This leads to insights into the behaviour of the person with dementia and its causes (outcome), raises awareness to consider the necessity of each care action at a specific moment (outcome), and leads to a shift from external towards internal attribution by team members (outcome).

However, participants felt that these changes were less prominent if team members were absent from feedback sessions.

*“Everyone is invited to join the sessions, and unfortunately, […] very few colleagues come back to watch the recordings. I don’t know why. (The result is that) next time they complain again because they were hit again.”* (Interviewee 2, nurse)

Context-Mechanism-Outcome configuration V

If team members are not motivated to attend feedback sessions (context) they will not become aware of (1) who the person with dementia is and (2) their part in the interaction (mechanism), which can lead to non-improved verbal and nonverbal communication between team members and the person with dementia (outcome), and escalating behaviour of the person with dementia (outcome).

#### 3.1.2 Safety and trust within the team

Participants also mentioned that they felt that safety and trust play a vital role in the potential results of feedback sessions and practice assignments.

*“If there is much collaboration and safety within the team, VIO is more effective. Because then team members provide more feedback to each other, and they communicate more easily about the practice assignments in between sessions.”* (Interviewee 4, VIO counsellor)

Moreover, safety and trust within the team were experienced to have an influence on team alignment. The feedback sessions offered a space to share and create a consistent approach.

*“No one is around 24/7. So you have to do it together, and for the resident it also is most pleasant if there is an unambiguous approach.”* (Interviewee 3, certified nurse assistant)

*“Each trajectory I’ve done so far, the team wasn’t aligned. […] Through watching the footage together, the team grew closer, and decided to create a consistent approach for that resident.”* (Interviewee 1, VIO counsellor)

Context-Mechanism-Outcome configuration VI

The feedback sessions facilitate the care team gaining shared awareness of their individual and cooperative stances (mechanism), especially if the collaboration between care team members is high (context). This leads to the care team being more aligned (outcome) and adopting a consistent approach (outcome).

#### 3.1.3 Team members with an exemplary role

Both VIO counsellors and team members mentioned how role models could influence the outcomes of VIO. The counsellors indicated the importance of identifying team members with an exemplary role. They described that a positively oriented role model could influence the other team members to open themselves up to VIO as well. Not only did this help in securing a positive experience with VIO for the care team, it also increased the willingness of the team to participate in a succeeding VIO trajectory.

*“I really tried my best to get VIO back in the unit. I asked the team who wanted to help me shape this next trajectory and we made clear agreements on who would participate. That way, we finished a VIO trajectory in a positive manner, and now we’re working on the third.”* (Interviewee 3, certified nurse assistant)

Moreover, participants felt that exemplary roles play a part within video footage as well, as they can motivate team members to be recorded and also to practice with the assignments.

*“Sometimes care team members are a bit embarrassed. If they get the chance to experience it by a colleague that is willing to be recorded, there is a chance that they will become willing as well.”* (Interviewee 6, VIO counsellor)

*“I can learn from it. I watch, and I know how I do it, but because I see how someone else does it more successfully, I can try that too next time. It’s no guarantee but you can continue to learn from each other how to care for a resident.”* (Interviewee 2, nurse)

Context-Mechanism-Outcome configuration VII

The footage in the feedback session shows how a colleague handled a situation. In line with whether the recorded colleague has an exemplary role (context), this footage increases both the motivation to practice (outcome) and the motivation to be filmed (outcome) through identification with a colleague (mechanism).

#### 3.1.4 Expectations of VIO

Several interviewees mentioned that team members can be reluctant to participate in VIO, due to their incorrect expectations from the intervention.

*“Colleagues often need to gain trust, especially in the beginning or if it’s their first time, that ‘VIO isn’t meant to tear me down or point out what I’m doing wrong’. It is meant to see what you inadvertently are doing well, and what another colleague can learn from it, that’s what VIO is. But not everyone knows, and that makes it scary to be filmed for them.”*’ (Interviewee 3, certified nurse assistant)

*“A strength of VIO is that you can immediately see what qualities someone uses. […] And what is not done well, is never mentioned, the focus always is ‘what are you doing well?’*. (Interviewee 11, certified nurse assistant)

Context-Mechanism-Outcome configuration VIII

During VIO, the VIO counsellor focuses on positive reinforcement of communicative elements that work, which creates trust in the team member (mechanism), especially if the team member is scared that VIO will have a negative focus (context). This ensures that the team member is willing to participate in future implementations of VIO (outcome).

### 3.2 The VIO counsellor’s personal approach

The interviewees identified various ways in which the VIO counsellor undertakes additional efforts to increase the chance of positive outcomes of the trajectory. An example mentioned was to opt for an additional information session if VIO is new to the organization or if a lot of resistance can be expected. Additionally, the interviewees remarked that the VIO counsellor could invest in mutual trust with the care team, which enhances the sense of ease experienced by the care team.

*“First, I invest in the relationship with the person I’ll be recording through a small conversation beforehand. I continuously stress that it’s not about right and wrong, but about understanding what happens. If I don’t do it, afterwards people say ‘oh, I am so nervous’, yet if I do it people say that they quickly felt at ease around me.”* (Interviewee 10, VIO counsellor)

Moreover, the interviewees noted the option of the VIO counsellor to take an active part in ensuring retention of the lessons learned, by organizing a final multidisciplinary feedback session.

*“With every case I invest in inviting different employees; the psychologist, the person-centred care staff member, the occupational therapist. I find it important to assure for the future. I come and go with this VIO trajectory. But if I am gone, I want the psychologist or others to be able to get back to it together, multidisciplinary.”* (Interviewee 5, VIO counsellor)

Context-Mechanism-Outcome configuration IX

The VIO counsellor organises a final session for the care team, and invites as many disciplines as possible (i.e. psychologist, elderly care physician) to join (context) which leads to a growing, multidisciplinary feeling of responsibility for preserving the practical tools (mechanism). This in turn means that someone within the organisation can take over the coaching role of the VIO counsellor (outcome).

### 3.3 Organisational conditions

Many respondents described organisational conditions that they consider relevant for how VIO works. Organisational conditions were of importance throughout the entire intervention process. Lack of both permanent and temporary staff, high sick leave and unclarity on whether VIO hours were reimbursed as working hours, were mentioned as reasons for team members to not attend feedback sessions.

*“I don’t think VIO yields the same for all team members. It depends on how many feedback sessions people attend. Ideally, I start with a group and that group is consistently present. Well, in practice that isn’t the case due to work schedules, high sick leave, lack of permanent and temporary staff.”* (Interviewee 4, VIO counsellor)

*“You hope to get the same team members (every feedback session). But currently it’s hard to gather groups in long-term older adult care. Nearly no real teams exist anymore. There are many freelancers working everywhere. And their work is alright, but they’ll never really join a team.”* (Interviewee 8, VIO counsellor)

*“Honestly, few people come back for a feedback session on their day off. So the organisation should provide financial reimbursement to carry a VIO trajectory together. So that we get the hours paid if a feedback session takes place during our time off, or that we assure that as many permanent team members as possible work on the day of the feedback session.”* (Interviewee 11, certified nurse assistant)

*“I think an organisation needs to see VIO’s importance. If the organisation is open to setting apart time, space and expertise this has an influence for the residents, for us, to be able to handle them, and more quickly start a VIO trajectory.”* (Interviewee 2, nurse)

Context-Mechanism-Outcome configuration X

If the organisation sees the importance of VIO (context), the organisation will be more willing to facilitate it (mechanism), ensuring VIOs timely execution (outcome).

Additionally, respondents mentioned the role of the manager in the success of VIO.

*“It has a lot to do with the manager. Whether they convey that VIO deserves time and attention. Or a team leader, I always ask them to attend the feedback session to give compliments to the team, and hear how tough it can be. If your manager supports you a little every day, it makes it easier.”* (Interviewee 9, VIO counsellor)

Context-Mechanism-Outcome configuration XI

If managers are unaware of what VIO is or if they do not support the intervention although it is implemented (context), team members will not feel supported (mechanism) and VIO will be less effective (outcome).

### 3.4 The role of the family of people living with dementia

Interviewees mentioned that family has three main ways to connect with VIO. They can read reports on the feedback sessions in the online patient portfolio, look at all recorded footage in a separate feedback session after VIO for the care team has finished, or they can discuss the situation with the care team. The role of the family was described as follows:

*“Most families read along in the online patient portfolio, in which it is reported what we’ve done and seen and what the practice assignments are. Some families initiate a conversation on these reports with their own questions and others have to be directed towards the reports and explained what they mean.”* (Interviewee 2, nurse)

*“I often think that family feels like we’re working together as a result of VIO. It’s a bit about being heard and us as professionals giving guidance to the family. Because they often have ignorance regarding dementia, but it also helps the family to accept. […] I was just thinking, the VIO is ongoing and family is updated but maybe family could be more formally involved because due to staff shortage we need to ask more of the family. So, we need them to work with us to improve the quality of care for our residents. But also to give them the feeling that they can care for their loved one as well as we do.”* (Interviewee 11, certified nurse assistant)

Context-Mechanism-Outcome configuration XII

Family willing to discuss the situation with the care team (context), is open to the practical tools when team members share these (mechanism). This can lead to improved verbal and nonverbal communication between family and the person with dementia (outcome), an improved relationship between family and the person with dementia (outcome), grown trust between family and the care team (outcome), more collaboration between family and the care team (outcome), and a decrease in distress behaviour of the person with dementia (outcome).

## 4 Discussion

To our knowledge, this is the first study exploring how video feedback interventions – and more specifically a Dutch video feedback intervention called ‘VIO’ – work in nursing homes, for whom, and under which circumstances. We found that VIO can improve verbal and nonverbal communication with persons living with dementia that exhibit distress behaviour. VIO's working mechanism seems to be increasing awareness of team member’s contribution to the interaction with the person living with dementia. We propose that this awareness follows from attendance to feedback sessions with reflectively watching video footage and stimulating questions asked by the VIO counsellor, and practising with tailored assignments. Safety and trust within the care team, team members with an exemplary role who are positively oriented towards VIO, and trust between the VIO counsellor and the care team appear to be facilitators of VIO outcomes. Organisational restraints like lack of staff, high sick leave in care teams, and lack of managerial support seem to be barriers. Furthermore, our results suggest that improved verbal and nonverbal communication between the family and the person living with dementia may be achievable if the family is open to the practical tools resulting from VIO, when the care team shares these with the family. Last, our findings indicate that the improved verbal and nonverbal communication with the person living with dementia can form the starting point for positive changes on other possible outcomes like distress behaviour, the relationship, control over the situation by team members, and collaboration between the care team and family.

Our findings on the shift in focus of the team members from the behaviour of the person with dementia towards team members’ own contribution to the interaction can be conceptualised in attribution theory (Cook and Artino, 2016). By literally seeing how their actions contribute to the interaction, and being asked stimulating questions, team members may be encouraged to take responsibility for the interaction and no longer frame successes of their colleagues as ‘a special connection’. They can begin to realise how they contribute to the interaction and that they, therefore, are also able to influence it. In doing so, they possibly start attributing more internally. Through the stimulating questions moreover, they can gain more understanding of the person living with dementia, possibly resulting in a more positive attitude towards this person. The possible side effect of VIO, namely a decrease in frequency and severity of distress behaviour, is in line with other studies showing that attitudes and skills of care staff has been related to a decrease in distress behaviour in dementia (Gerritsen et al., 2019; van Voorden et al., 2023).

The interviewees emphasised the importance of safety, shared awareness, and learning together as a team. In accordance with this, a study focusing on a reflective psycho-educational intervention in dementia care demonstrated that emotional support in sharing anxiety and work-related issues helped care staff in developing positive relationships among each other, and improved care staff’s motivation to change (Barbosa et al., 2017). This suggests that although VIO aims to improve the interactions and attunement, and – through those – the care relationship between the person living with dementia and the care team, it might actually rather lead to improved relationships within the care team. Moreover, the development of positive relationships among team members might facilitate overcoming incorrect expectations of VIO. An explanation is that positive relationships with a team member with an exemplary role can be linked to the concept of modelling in self-efficacy theory. Modelling, in essence, means that a learner takes the outcomes of others’ actions into consideration while learning (Cook and Artino, 2016). In case of VIO this means that a team member who might be anxious to participate in VIO, can be persuaded by the successful example of another team member to participate and reach success through VIO. Our results, for example, showed that if team members with an exemplary role agreed to be recorded, this could motivate others to agree to be recorded as well. In line with this, an explorative study into a video-based intervention in a care organisation for people with autism spectrum disorder discovered that staff initially thought the intervention would focus on the negative rather than positive practice. However, enthusiasm from earlier involved employees encouraged more staff to try the intervention (Hall et al., 2016). Likewise, our results indicate that a VIO counsellor who has a positive focus and addresses anxiety and incorrect expectations revolving around being recorded, can motivate care staff to participate in VIO.

Daily work demands and absence of managers’ support have previously been identified as challenges in implementing video feedback intervention (Hall et al., 2016). This is in line with our findings, as they can be barriers to attending VIO’s feedback sessions. Nevertheless, these organisational restraints are a modern-day reality and cannot always be eliminated (van Teunenbroek et al., 2020). As care staff’s ability to participate has been recognised as a necessity (see also Alnes et al., 2013), other strategies for enhancing feedback session attendance should be explored. For example, not all video feedback interventions require on-site feedback sessions as part of their protocol. Noordman et al. (2014) studied a web-based video feedback intervention to enhance communication in practice nurses. Their results suggest that web-based video feedback can also improve communication skills. Moreover, a video-based training module in communication has shown promising results (Lenoir et al., 2025). Both would allow more flexibility in scheduling hybrid feedback sessions, although it would require an investment in digital literacy of the care team, as lack of digital skills have been identified as a barrier to videoconferencing (Beattie et al., 2025). Alternatively, a kick-off meeting with a video-based training module may be included in the VIO protocol at the start to enhance general knowledge of care staff. Yet, as VIO in the nursing home is team-oriented and aimed at actual interactions, diligent research is necessary to ensure that hybrid feedback sessions or pre-existing training modules do not result in unintended negative outcomes.

Moreover, we found that the improved verbal and nonverbal communication of team members can apply to both the person living with dementia who was the focus of VIO, as well as to other people in the unit living with dementia. This suggests that lessons learned through VIO can improve overall verbal and nonverbal communication with people living with dementia and that, through VIO, team members can improve the quality of dementia care within a specific organisation. Similarly, a feasibility study into an observational tool suggests that reflective watching was a strong means to increase awareness and understanding of the person living with dementia, thereby improving individual care staff practice as well as organisation wide (Surr et al., 2023). Furthermore, a study comparing problem-based training to team-based training in nursing homes for people with dementia found that the team-based arm tended to describe more anticipatory responses (Como et al., 2025), which could explain our finding as well.

Currently, the support request of the care team is the focus of VIO in nursing homes. This leaves the family with limited opportunities to get involved in VIO in nursing homes. However, in line with societal developments in which the family increasingly is seen as a collaborative care partner, the time seems right for the family to be more formally involved, as one of the interviewees also suggested. Moreover, we found that VIO may potentially strengthen the collaboration between the care team and family, as they can learn about caring for the person living with dementia from each other, since they both are “experts” on caring for the person living with dementia, albeit with a different perspective (Como et al., 2025; Fisher et al., 2025; Hovenga et al., 2024; Smebye and Kirkevold, 2013). Studies into video feedback interventions at home indicate that primary caregivers benefitted from video feedback interventions (Berwig et al., 2020; Gerritsen, Koopmans, et al., 2019; Shaw et al., 2020). Similar to our results, acquiring insight into the person with dementia and their behaviour, and better contact with their relative were mentioned as outcomes during the interviews. However, not all primary caregivers were willing to participate in the intervention. More or less the same limiting factors in willingness to participate were mentioned for this as in our study: unfamiliarity with video feedback, perceived threat of the camera and an unfamiliar person, and the care team member’s feeling of acting differently in front of the camera (Gerritsen, Koopmans, et al., 2019). Apart from these potential barriers, more formally involving the family also raises some points of concern that need to be considered, such as establishing a more formal collaboration between the care staff and family (Tasseron-Dries et al., 2023), ensuring safety and trust between the care staff and family and considering the possibility of organising separate feedback sessions for family and the care team because of possible differences in appraisal of the behaviour shown in the recorded footage (Fisher et al., 2025). Future research should explore which strategies are viable to overcome incorrect expectations of both family and care team with regards to VIO and how to best create and strengthen collaboration between the care team and family, guaranteeing safety and trust between them.

### 4.1 Strengths and limitations

A strength of this study is the methodology of choice: realist evaluation. The focus of realist evaluation on generative causation ensured that we uncovered in-depth explanations for how VIO works, for whom and in which circumstances. Thereby, we added to the development of program theory of video feedback interventions. Furthermore, throughout this study, diligent attention was paid to explanatory adequacy, relevance and rigour, in line with realist evaluation. Explanatory adequacy was upheld through immersion in the data and refinement of Context-Mechanism-Outcome configurations. Criteria of relevance and rigour were applied in selection of interviewees and data analysis. This not only advances knowledge on video feedback interventions and how they work, but also provides insights that can be applied in practice. As a next step, a realist evaluation of multiple VIO trajectories would be advisable. In that evaluation, the discovered Context-Mechanism-Outcome configurations can be tested and refined. Additionally, this evaluation could include a comparison of VIO against other commonly used interventions to develop knowledge regarding what works best, for whom, and under which circumstances. Moreover, study designs from other paradigms, like randomized controlled trials, could prove useful to further investigate VIO’s effectiveness.

An important limitation to be noted is the possibility of bias. Firstly, the interviewees were recruited through the network of the project group. It is likely that the project group members invited interviewees with positive attitudes towards VIO. Moreover, people with positive attitudes towards VIO are also more likely to partake in an interview, than those with negative attitudes. However, we purposely chose our interviewees based on their experience with VIO, and the interview topic guide explicitly focused on both successful and unsuccessful VIO trajectories. As a result of this both positive and negative experiences have been included in this study. Moreover, the fact that we included the perspective of two stakeholders (VIO counsellors and team members), adds to the data sources being complementary and confirmatory.

A second constraint of our study is that two of the stakeholders (i.e. people living with dementia and family) were not included as interviewees. Due to the stage of the condition, the people living with dementia that were eligible for VIO were no longer able to be interviewed. Although this limitation cannot be overcome, it remains important to take this into account. Additionally, family members were not interviewed either. Considering the current limited role of family in VIO in nursing homes and the fact that the primary focus of VIO in nursing homes currently is the care team, we do not expect this to have greatly influenced our results. Yet, as family is increasingly seen as a collaborative care partner, it be interesting to explore how they could be more formally involved.

## 5 Conclusions

In conclusion, by interviewing VIO counsellors and care team members and taking on a realist perspective, this study has found that VIO is considered to improve verbal and nonverbal communication with the person with dementia, and can work because team members become aware of their contribution to the interaction with the person with dementia. We found several factors – namely team, organisational and counsellor – explaining how VIO can work, for whom, and in which circumstances. Future research should focus on testing and refining the discovered Context-Mechanism-Outcome-configurations in practice because this will enhance theory on how video feedback interventions work. Moreover, it is recommended that daily practice explores how to increase the role of family and how to overcome incorrect expectations. Ultimately, this can reduce distress behaviour in nursing homes, improve quality of care for people living with dementia and decrease both formal and informal caregiver distress.

## Funding

This work was supported by the Netherlands Organisation for Health Research and Development (ZonMw), grant number [639003918].

## CRediT authorship contribution statement

**Stephanie Timmermans:** Writing – review & editing, Writing – original draft, Visualization, Methodology, Investigation, Formal analysis, Conceptualization. **Annette Plouvier:** Writing – review & editing, Writing – original draft, Visualization, Supervision, Formal analysis. **Anne Kolmans:** Writing – review & editing, Visualization, Formal analysis. **Mandy Wijnen:** Writing – review & editing, Methodology, Investigation, Formal analysis, Conceptualization. **Anke Persoon:** Writing – review & editing, Supervision, Methodology, Formal analysis, Conceptualization. **Annemiek Bielderman:** Writing – review & editing, Methodology, Formal analysis, Conceptualization. **Cécile Boot:** Writing – review & editing, Visualization, Supervision, Methodology, Formal analysis, Conceptualization. **Debby Gerritsen:** Writing – review & editing, Visualization, Supervision, Methodology, Formal analysis, Conceptualization.

## Declaration of competing interest

The authors declare that they have no known competing financial interests or personal relationships that could have appeared to influence the work reported in this paper.
